# Call for a unified approach to Monkeypox infection in pregnancy: Lessons from the COVID-19 pandemic

**DOI:** 10.1038/s41467-022-32638-w

**Published:** 2022-08-26

**Authors:** Asma Khalil, Athina Samara, Pat O’Brien, Shamez Ladhani

**Affiliations:** 1 Fetal Medicine Unit, St George’s Hospital, St George’s University of London, London, UK; 2 Vascular Biology Research Centre, Molecular and Clinical Sciences Research Institute, St George’s University of London, London, UK; 3 Department of Women’s and Children’s Health, Karolinska Institutet, Stockholm, Sweden; 4 Astrid Lindgren Children’s Hospital, Karolinska University Hospital, Stockholm, Sweden; 5 The Royal College of Obstetricians and Gynaecologists, London, UK; 6 University College London Hospitals NHS Foundation Trust, London, UK; 7 Paediatric Infectious Diseases Research Group and Vaccine Institute, Institute of Infection and Immunity, St George’s University of London, London, UK; 8 Immunisation and Countermeasures Division, Public Health England, London, UK; 9 British Paediatric Surveillance Unit, Royal College of Paediatrics and Child Health, London, UK

**Keywords:** Viral infection, Epidemiology, Public health, Vaccines

## Abstract

In the current monkeypox outbreak, vaccination and treatment of pregnant women are recommended only if the benefits outweigh risks, but the extremely sparse data available limit evidence-based recommendations. We must facilitate a unified consensus approach to rapidly collect robust data. Two years into the COVID-19 pandemic, monkeypox has emerged as yet another challenge to the mother-fetus dyad, potentially placing both at risk, if exposed.

## Epidemiology

The multi-country monkeypox outbreak that began in early May 2022 is the first for which the source cannot as yet be directly traced back to Africa, where this disease is endemic. It was declared a public health emergency of international concern (PHEIC) by the World Health Organization (WHO) on July 23. Laboratory investigations have confirmed that this outbreak is caused by the West African clade of monkeypox (https://virological.org/t/first-draft-genome-sequence-of-monkeypox-virus189associated-with-the-suspected-multi-country-outbreak-may-2022-confirmed-case190 in-portugal/799), which was previously documented to have a case fatality ratio of around 1%. As of 22 July 2022, more than 16,500 confirmed cases have been reported worldwide (https://ourworldindata.org/explorers/monkeypox?facet=none&pickerSort=desc&pi ckerMetric=total_confirmed_by_confirmation&Metric=Confirmed+cases&Frequen cy=Cumulative&Shown+by=Date+of+confirmation&country=~OWID_WRL), with numbers continuing to rise. As yet, there are 5 reported deaths; this apparently lower case fatality rate (CFR) may be due to several factors. Under-reporting of mild cases in Africa may lead to overestimation of the CFR there; medical care may be better and delivered earlier in high income countries (HICs); or this may be a less virulent strain of the virus.

As was the case for COVID-19 until recently, there are no approved treatments or vaccines for monkeypox in pregnancy, highlighting an urgent need for a systematic approach to the prevention, investigation and management of pregnant persons exposed to monkeypox. Given the rarity of infection and the high-risk population involved, clinical trials, including randomized controlled trials, are unlikely to represent a realistic option for swiftly collecting data in the context of a rapidly spreading global outbreak.

## Limited information on monkeypox in pregnancy

Data on monkeypox infection in pregnancy are restricted to very few case reports and small case series in endemic regions. A non-laboratory-confirmed case of monkeypox in a pregnant woman (~24 weeks’ gestation) in the Democratic Republic of Congo (DRC), was associated with premature birth, with the infant exhibiting a skin rash consistent with monkeypox and dying of malnutrition 6 weeks later^1^. An observational study of 222 symptomatic infected hospitalized individuals, also in the DRC, included 4 pregnant women, 3 of whom experienced fetal demise^2^. Two had first trimester miscarriages while the third patient had moderately severe disease at 18 weeks’ gestation and suffered an intrauterine fetal demise^2^. The fourth woman delivered a healthy baby at term. There was no testing of pregnancy tissue for the miscarriages. In the case of the intrauterine fetal death, virological, histological, and serological evidence were consistent with vertical transmission, with the fetus demonstrating features of monkeypox infection, including the characteristic rash.

## Prophylaxis and treatment

**Fig. 1:**
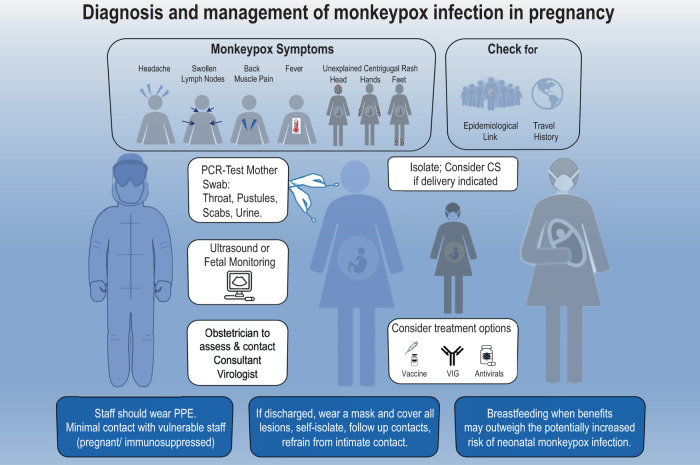
Diagnosis and management of monkeypox infection in pregnancy. Pregnant women may present with an unexplained centrifugal rash (head, hands and feet) or a rash on any part of the body, and report one or more classical symptom(s) of monkeypox infection, including intense headache, lymphadenopathy, arthralgia and/or backache and fever (>38.5 C). They may have an epidemiological link to a confirmed or probable case of monkeypox, or travel history to West or Central Africa in the 21 days before symptom onset. Staff wearing personal protective equipment (PPE) should take a sample for polymerase chain reaction (PCR) (throat, pustules, scabs or urine) for monkeypox virus. The patient should be masked, lesions covered, and isolated; monkeypox treatment options should be discussed, and the consultant virologist contacted. The obstetrician should assess the fetus (ultrasound/fetal heart rate monitoring) and mother and consider cesarean section (CS) if delivery is indicated or the woman is in labour. Staff examining patients should wear PPE at all times; contact with vulnerable staff (pregnant/ immunosuppressed) should be minimized. If patients are discharged, they should wear a mask and cover all body lesions, self-isolate, refrain from intimate contact, and their contacts should be followed up. After delivery, breastfeeding should be considered when benefits may outweigh the potentially increased risk of neonatal monkeypox infection (for example in low- and middle-income countries, breastfeeding may carry greater benefit to the baby than the potential risk of neonatal monkeypox infection). VIG: vaccinia immune globulin.

Monkeypox infection in pregnancy potentially places two individuals (mother and baby) at risk, and the current Centers for Disease Control and Prevention (CDC) guidance suggests that pregnant and breastfeeding women ‘should be considered for treatment following CDC consultation’ (https://www.cdc.gov/poxvirus/monkeypox/clinicians/treatment.html#Tecovirimat) (Fig. 1). The antivirals cidofovir, brincidofovir and tecovirimat, and the smallpox vaccine or vaccinia immune globulin (VIG) may be considered for adults. Tecovirimat is not licensed for the treatment of monkeypox in pregnancy, cidofovir is recommended only if the pregnant woman is severely ill. According to the US Food and Drug Administration (FDA), an alternative treatment to brincidofovir should be used in pregnancy, if feasible^3^. VIG is an antibody cocktail purified from the blood of individuals previously immunized with the smallpox vaccine. Although little is known about its safety or efficacy in pregnancy, other immunoglobulins have been studied extensively and generally found to be safe in pregnancy.

The third-generation smallpox vaccine, MVA-BN, is 85% effective against monkeypox.

Post-vaccination follow-up of fewer than 300 pregnant women did not identify an increase in adverse outcomes^4^, but MVA-BN is not yet approved for use against monkeypox in pregnancy. This vaccine, however, contains non-replicating virus and animal studies have not identified any adverse fetal effects, but the general advice remains that MVA-BN vaccine should be used during pregnancy only if the benefits, in terms of protection of both mother and baby, of preventing monkeypox likely outweigh any potential unknown risks associated with the vaccine. On the other hand, MVA-BN is considered safe during breastfeeding and should be offered to breastfeeding women with significant exposure to the virus after discussing the risks of monkeypox to them and their infants (Fig. 1).

## Pregnant women in clinical trials

Historically, pregnant women have been actively excluded from clinical trials due to physiological and ethical concerns. Many trials advise participants not to fall pregnant following recruitment and, if they do, exclude them from further participation. Compounds and medications that cross the placenta could potentially affect fetal development, growth, or irreversibly alter organ function, potentially resulting in significant harm to the unborn fetus. The numerous changes in maternal physiology, along with potentially inconsistent evidence regarding teratogenicity and toxicity profiles between animal and human pregnancies should also be considered. The thalidomide tragedy has set back research into pregnancy therapeutics, dissuading pharmaceutical companies and regulators from including pregnant people in trials of new therapeutics. The effect of that tragedy on trial design persists, with consequent delays and potentially denying generations of pregnant women and their babies the benefits of new therapeutics.

## Pregnant women in COVID-19 clinical trials

For much of the COVID-19 pandemic, pregnant women were excluded from preventive and therapeutic trials^5^ although there were some exceptions, such as the UK RECOVERY Trial (https://www.recoverytrial.net/files/recovery-in-pregnant-women-for-hcp-v15-1-2021-12-22.pdf) (https://clinicaltrials.gov/ct2/show/NCT04765384) and others^6^. Consequently, effective treatments such as corticosteroids for severe COVID-19 patients which was implemented into clinical practice in the UK in June 2020, remain less frequently used in pregnant than non-pregnant women^7^.

Pregnant women were also excluded from phase III COVID-19 vaccine trials which irrevocably delayed the accumulation of evidence on safety and efficacy in pregnancy, and significantly contributed to vaccine hesitancy when the vaccines were later recommended by national bodies, such as the National Immunization Technical Advisory Groups (NITAGs), outside marketing authorization for pregnant people, including pregnant healthcare workers.

A systematic approach to rapidly collecting post-implementation safety and effectiveness data on COVID-19 vaccines after they were recommended for pregnant people, would have helped to reassure this high-risk population of their safety and efficacy much sooner. Observational data have now corroborated the findings of the randomized controlled trials in adults and shown mRNA vaccination to be highly effective at preventing SARS-CoV-2 infection, thus protecting both the mother and fetus^8,9^. Studies have also showed that mRNA vaccines do not increase the risk of miscarriage, preterm birth, low birthweight, maternal or neonatal intensive care unit admission, fetal death, fetal abnormality or pulmonary embolism^8^. Yet to this day, tackling COVID-19 vaccine hesitancy in pregnant people remains a major challenge because of the fear and mistrust resulting from lack of safety and efficacy data in this high-risk group in early clinical trials.

## International strategies and global registries

Delays in treatment and immunization recommendations have placed pregnant women and their babies at unnecessary risk of preventable maternal morbidity and mortality, with an increased risk of adverse pregnancy outcomes. Developing a systematic consensus approach for all pregnant people with standardised data collection would allow for rapid evaluation of preventive and therapeutic measures for pregnant people exposed to monkeypox in near real-time. By establishing international strategies (such as the International Severe Acute Respiratory and emerging Infection Consortium (ISARIC)), a standardised approach could be implemented and evaluated in multiple regions affected by the outbreak, which could rapidly provide real world data on efficacy according to the gestation and could include neonatal outcomes.

For pregnant people, a similar set-up as the COVAX COVID-19 Maternal Immunization Working Group (MIWG https://epi.tghn.org/covax-overview/clinical-science/maternal240immunization/vaccine-safety-mi/10), which developed specific criteria to evaluate COVID-19 vaccine candidates in pregnant people, could be used. This group encompasses, among others, clinical medicine and research, pharmacovigilance, vaccine safety, regulatory affairs, and ethics, and prepared a roadmap for study design, data availability, communication, and regulatory considerations to facilitate vaccine access for pregnant individuals worldwide to combat COVID-19.

## Lessons to be learned

The current advice is that pregnant women should be vaccinated or treated for monkeypox if the benefits outweigh the risks. The extremely limited data available on safety or efficacy, however, hinder evidence-based care for this high-risk group. Learning from the COVID-19 pandemic and planning ahead, we must adopt not only a consistent unified approach, but also the infrastructure and global registries for linked pregnancy and vaccine surveillance systems to rapidly collect and disseminate safety and other data during the Emergency Use Authorization (EUA) and post-EUA approval periods. Such registries and infrastructure are currently mostly restricted to HICs, but the data collected in affected HICs would benefit pregnant women and their infants in both HICs and low- and middle-income countries (LMICs).

At the same time, however, it must be recognized that had appropriate investment been made to implement systems to prevent, treat and gather robust data in LMICs where monkeypox has been endemic for many decades, not only would we have greatly benefitted the health of those populations, but we would also now have far more evidence to inform prevention and treatment during the current outbreak. It is also critical that, once vaccines and treatments are found to be safe and effective against monkeypox, they are made available to LMICs, where there are far more cases.

The recent COVID-19 pandemic and the current monkeypox outbreak should be used as a blueprint to develop and implement a consistent unified approach to prevention, investigation, and management of rare diseases not only for pregnant people, but also for other vulnerable and high-risk groups, such as children and immunocompromised populations, in both HICs and LMICs.
